# Mechanistically detailed systems biology modeling of the HGF/Met pathway in hepatocellular carcinoma

**DOI:** 10.1038/s41540-019-0107-2

**Published:** 2019-08-16

**Authors:** Mohammad Jafarnejad, Richard J. Sové, Ludmila Danilova, Adam C. Mirando, Yu Zhang, Mark Yarchoan, Phuoc T. Tran, Niranjan B. Pandey, Elana J. Fertig, Aleksander S. Popel

**Affiliations:** 1 Department of Biomedical Engineering, Johns Hopkins University School of Medicine, Baltimore, MD USA; 2 Department of Oncology, Division of Biostatistics and Bioinformatics, Sidney Kimmel Comprehensive Cancer Center, Johns Hopkins University, Baltimore, MD USA; 3 The Sidney Kimmel Comprehensive Cancer Center, Johns Hopkins University School of Medicine, Baltimore, MD USA; 4 Department of Radiation Oncology and Molecular and Radiation Sciences, Sidney Kimmel Comprehensive Cancer Centre, Johns Hopkins University School of Medicine, Baltimore, MD USA; 5 Department of Medical Oncology, Sidney Kimmel Comprehensive Cancer Centre and Department of Urology, The Brady Urological Institute, Johns Hopkins University School of Medicine, Baltimore, MD USA; 6 Department of Applied Mathematics and Statistics, Johns Hopkins University, Baltimore, MD USA

**Keywords:** Computer modelling, Cancer

## Abstract

Hepatocyte growth factor (HGF) signaling through its receptor Met has been implicated in hepatocellular carcinoma tumorigenesis and progression. Met interaction with integrins is shown to modulate the downstream signaling to Akt and ERK (extracellular-regulated kinase). In this study, we developed a mechanistically detailed systems biology model of HGF/Met signaling pathway that incorporated specific interactions with integrins to investigate the efficacy of integrin-binding peptide, AXT050, as monotherapy and in combination with other therapeutics targeting this pathway. Here we report that the modeled dynamics of the response to AXT050 revealed that receptor trafficking is sufficient to explain the effect of Met–integrin interactions on HGF signaling. Furthermore, the model predicted patient-specific synergy and antagonism of efficacy and potency for combination of AXT050 with sorafenib, cabozantinib, and rilotumumab. Overall, the model provides a valuable framework for studying the efficacy of drugs targeting receptor tyrosine kinase interaction with integrins, and identification of synergistic drug combinations for the patients.

## Oncology: Scientists predict efficacy of cancer therapies

Mathematical modeling can be used to predict the efficacy of drug therapies in liver cancer for individual patients. A team lead by Mohammad Jafarnejad at Johns Hopkins University developed a mathematical model of cellular signaling in liver cells that can predict drug interactions affecting the growth pathways in liver cells found to be responsible for tumor formation in liver cancer. The model was calibrated based on available data and was able to predict the efficacy of common therapies and predicted a synergy for a combination of therapies. Overall, the mathematical modeling allowed us to study the differences in efficacy between different drugs and the efficacy of combining drugs. With the expected increase in the availability of patient-specific data, the methods developed here can be used to benefit individual patients by determining which therapies are most likely to be effective.

## Introduction

Hepatocyte growth factor (HGF) is an essential growth factor for liver regeneration,^1^ embryogenesis,^2^ and wound healing.^3^ HGF signaling through its receptor, Met (also known as c-Met), plays an important role in tumor invasion, metastasis, and angiogenesis,^4^ and is also identified as one of the resistance mechanisms to targeted therapies against both tumor growth and angiogenesis.^5^ HGF was discovered as a potent factor to induce cell migration, hence was called “scatter factor.”^6^ In embryogenesis, HGF/Met signaling results in epithelial-to-mesenchymal transition (EMT) in myogenic progenitor cells and migration of those cells over long distances.^2^ After partial hepatectomy, HGF activates Met on hepatocytes, resulting in cell cycle progression, cell migration, and regeneration.^7^ Met-ablated keratinocytes in the skin failed to contribute to wound repair in mice, emphasizing the critical role of Met-induced migration and proliferation in wound healing.^8^

The HGF/Met axis pathway represents an attractive therapeutic target for many cancers including hepatocellular carcinoma (HCC) because of its putative role in tumorigenesis and invasion. HCC is the most common primary liver cancer, accounting for 90% of all primary liver cancers.^9^ It is the second leading cause of cancer death around the world, and in the United States, death from HCC is rising faster than the rate of death from any other cancer. While early stages of HCC can often be treated with curative therapies or locoregional therapies, more than half of all patients with HCC develop advanced stage HCC and are eligible for systemic therapy.^9^ Approval of sorafenib (multi-kinase inhibitor of vascular endothelial growth factor (VEGF), platelet-derived growth factor (PDGF), and Raf kinases) was the first systemic therapy to conclusively demonstrate an improvement in overall survival in patients with advanced HCC.^10^ More recently, several other agents have demonstrated clinical activity in HCC and have been incorporated into and adopted by major HCC guidelines, including lenvatinib^11^ (multi-kinase inhibitor of VEGF, fibroblast growth factor, and PDGF pathways) in frontline and cabozantinib^12^ (multi-kinase inhibitor of Met and VEGF pathways), regorafenib^13^ (multi-kinase inhibitor of VEGF and angiopoietin receptor pathways), and ramucirumab^14^ (anti-VEGF receptor 2 antibody) in the second-line setting. In contrast to the success of cabozantinib, rilotumumab (an anti-HGF monoclonal antibody) has not shown efficacy and its development was halted due to increased mortality.^15^ Additionally, immunotherapeutic antibodies against programmed cell death protein 1 (PD-1) (nivolumab and pembrolizumab) were recently granted accelerated approval in HCC on the basis of encouraging activity in earlier stage clinical trials.^16^ Although the recent wave of drug approvals for HCC is encouraging, patient outcomes have improved only modestly.^17^ There still remains a need for novel treatment strategies in terms of drug combination and biomarker development to ensure optimal utilization of the drugs for treatment of HCC.

Based on the crystal structure, HGF forms dimers,^18^ and binds to Met to promote dimerization and in turn autophosphorylation of the receptor at Tyr1234 and Tyr1235 that activates the intrinsic kinase activity, and phosphorylation at Tyr 1349 and Tyr 1356 that activates a docking site for adapter proteins.^19^ Phosphorylated Met (pMet) directly recruits adapter proteins such as Gab1, Grb2, and Src, which transmit the signal to two major downstream pathways of Akt and ERK (extracellular-regulated kinase).^19^ Gab1 recruits and activates phosphoinositide 3-kinase (PI3K) that converts PIP2 (phosphatidylinositol-4, 5-bisphosphate) to PIP3 (phosphatidylinositol-3, 4, 5-triphosphate), which goes on to phosphorylate Akt.^6^ Activation of ERK by the Met receptor proceeds through a cascade of kinases that begins with the phosphorylation of Grb2 and continues in order through SOS, Ras, Raf, and MEK (mitogen-activated protein kinase kinase), which ultimately activates ERK. While there is a chain of positive signals from Met activation to Akt and ERK phosphorylation, numerous feedback loops and crosstalk between the Akt and ERK arms of the pathway have caused drug development against these targets to be particularly challenging.^20^

The association of receptor tyrosine kinases (RTKs) with integrins has been shown to modulate the extent and magnitude of the RTK signaling.^21^ Met is shown to associate with various integrins such as α5β1,^22–26^ α6β4,^27^ and α3β1.^28^ The most studied of these interactions is the involvement of Met with the β1-subunit of α5β1 integrin. Bogorad et al.^29^ demonstrated that knockdown of αv and β1 integrins, using nanoparticle delivery of small interfering RNA (siRNA), resulted in reduced HCC progression and Met phosphorylation in vivo. Knockdown of β1 is shown to diminish liver regeneration through inhibition of Met and epidermal growth factor receptor (EGFR) signaling.^23^ In another study, Ju and Zhou^26^ indicated that inhibiting β1 integrin in combination with Met inhibition is necessary to overcome gefitinib (EGFR inhibitor) resistance in non-small-cell lung cancer.^26^ Association of fibronectin-bound α5β1 with Met is shown to lead to HGF-independent activation of Met.^22^ In a comprehensive study, Jahangiri et al.^24^ showed a physical interaction between β1 integrin and Met in vitro in breast cancer cell lines, and demonstrated that higher numbers of these complexes are found during invasive resistance in vivo in a glioblastoma model. β1 Integrin is also shown to co-internalize with Met and promote sustained ERK signaling using in vitro models of breast and lung cancer.^25^ All these studies point to the crucial role of α5β1 integrin in the regulation of HGF/Met signaling in cancer progression. Our previous work has shown that the disruption of integrin signaling using a novel extracellular matrix (ECM)-derived mimetic peptide drug, AXT050,^30^ inhibits HGF signaling through Met.^31^ AXT050 is also shown to be an antiangiogenic agent by reducing phosphorylation of VEGR2, IGFR, and PDGFR,^30^ to stabilize vessels by disrupting α5β1 signaling and relocating Tie2 receptors to the junction.^32^

In this study, we develop a mechanistically detailed systems biology model of the HGF/Met signaling pathway with detailed representation of the interactions of α5β1 integrin with Met on the cell surface that allowed us to explore the mechanism of action for AXT050 as monotherapy and in combination with other drugs targeting the HGF pathway (i.e., sorafenib, cabozantinib, and rilotumumab). We then adapted the calibrated model for individual patients based on the HCC data in The Cancer Genome Atlas (TCGA) and studied the efficacy of therapeutic strategies across the patients.

## Results

### Validated computational model captures main features of the HGF/Met pathway and interactions with α5β1 integrin

**Fig. 1:**
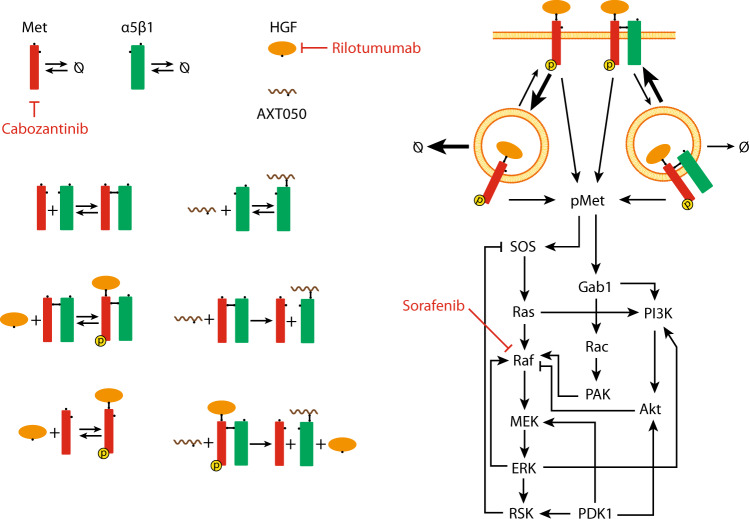
Diagram of the main molecular interactions implemented in the model. The diagram illustrates surface molecules and their interactions (on the left), as well as the intracellular downstream signaling to ERK (extracellular-regulated kinase) and Akt (on the right). Surface Met can bind hepatocyte growth factor (HGF) and get activated in the absence of α5β1 integrin that leads to rapid internalization and degradation, whereas integrin-bound Met has lower internalization/degradation and higher recycling rates. In addition to integrin-binding peptide (AXT050) that dissociates the Met/α5β1 complex, effect of anti-Met (cabozantinib), anti-HGF (rilotumumab), and Raf inhibitor (sorafenib) drugs were explored in this study

We constructed a computational model of HGF-mediated activation of the Met pathway as it refers to the important intracellular signals of Akt and ERK (Fig. 1). Additional interactions of Met receptor with surface α5β1 integrin were included to be able to capture the effect of the ECM-derived mimetic peptide, AXT050, on the regulation of this pathway (Fig. 1). Because the model was developed to investigate the effect of the HGF pathway on HCC cells, the literature was mined rigorously to be able to calibrate the model by only using datasets from hepatocytes^33^ or HCC cell lines.^30,34^ Additionally, the hepatocyte data from a study that quantified the abundance of proteins from an extensive and calibrated mass spectrometry dataset in isolated mouse primary liver cells^35^ (hepatocyte, hepatic stellate cell, hepatic sinusoidal endothelial cell, Kupffer cell, and cholangiocyte) were used in this study. The abundance of each protein was constrained from mass spectrometry data and a normalized time course of phosphorylation data was used as the input for pattern search algorithm (a global optimization technique) to fit the model to the totality of the gathered hepatocyte or HCC-specific data.

**Fig. 2:**
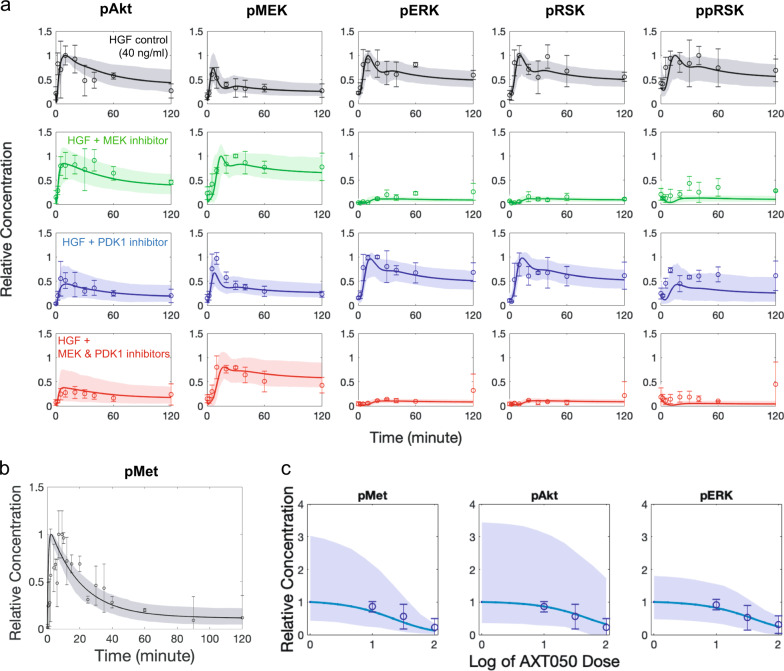
Calibration of the model by the available data in the literature. Global optimization using a pattern search algorithm was used to optimize all the model parameters using a consistent set of published data plotted here. **a** Primary data used for parameterization were obtained from the work of D’Alessandro et al.,^33^ who measured phosphorylation of Met, Akt, MEK (mitogen-activated protein kinase kinase), ERK (extracellular-regulated kinase) and RSK in vitro in primary mouse hepatocytes at multiple timepoints under treatment with hepatocyte growth factor (HGF) alone or in combination with various inhibitors (experimental data shown as mean ± SD, *n* = 3). Monte Carlo resampling technique was used to resample the experimental data and recalibrate the model to generate the confidence intervals of the model (modeling data is shown as baseline case and 95% range of the fitted simulations). **b** Phosphorylated Met (pMet) was also fitted to the experimental data from D’Alessandro et al.^33^ (experimental data are shown as mean ± SD, *n* = 3). **c** Additionally, phosphorylation of Met, Akt, and ERK were measured in our laboratory^30^ in HepG2 (human hepatocellular carcinoma cell line) after treatment with various doses of AXT050 peptide (experimental data are shown as mean ± SD, *n* = 3)

The calibrated model was able to capture the dynamics observed in the experimental data (Fig. 2). HGF treatment resulted in a rapid increase in pMet followed by its gradual decay (Fig. 2b). A similar pattern was observed for pAkt. In addition to HGF stimulation alone, data on pAkt, pMEK, pERK, and single and double phosphorylated RSK (pRSK and ppRSK, respectively) were available for HGF treatment along with MEK inhibitor, 3-phosphoinositide-dependent protein kinase-1 (PDK1) inhibitor, or both (Fig. 2a).^33^ This resulted in a rich dataset to assist in resolving the strength of feedback and crosstalk signals in the pathway. Inhibition of PDK1 resulted in a larger reduction in the pAkt compared to MEK inhibition in agreement with the experimental data (Fig. 2a). MEK inhibition resulted in an increase in the pMEK signal due to multiple and opposing feedback loops from ERK and RSK; this result was interesting and not obvious. Inhibition of MEK resulted in lower levels of pERK, pRSK, and ppRSK (Fig. 2a) that are directly downstream of the pMEK (Fig. 1). The globally fitted model correctly captured the more substantial contribution of the RSK to SOS negative feedback strength compared to the positive feedback from ERK to Raf, which resulted in the upregulation of pMEK after inhibition of MEK (Fig. 2). PDK1 inhibition had a minor effect on pMEK. The pMEK dynamics clearly demonstrated the need for extensive time-course data under a variety of treatments to reliably resolve the strength of competing feedback mechanisms. Monte Carlo resampling was used to quantify uncertainty in the model predictions (Fig. 2, confidence intervals) and calibrated parameters (Supplementary Fig. 1a). Moreover, local parameter sensitivities were used to distinguish practical identifiability of model parameters (Supplementary Fig. 1b). Considering multiple outputs of the model, all the model parameters appeared practically identifiable except the baseline production and degradation level of Met.

Dynamic interactions between Met and α5β1 integrin is a novel mechanism included in the model, and in addition to the data discussed so far, the AXT050 treatment data assisted in fitting the integrin interaction-related parameters. The model accurately captured the experimental data by Barbhuiya et al.^30^ based on treatment of HepG2 cells with HGF and increasing levels of AXT050 that resulted in progressive inhibition of pMet, pAkt, and pERK (Fig. 2c). The strength of sorafenib, a Raf inhibitor, was fitted to a dataset by Melas et al.^34^ from HCC cell lines, which showed reductions in pMEK and pERK directly downstream of the Raf, but not much changes in pAkt after 30 min. Largely, the model was able the capture the complex dynamics of Akt and ERK signaling pathways downstream of the HGF/Met on hepatocytes.

**Fig. 3:**
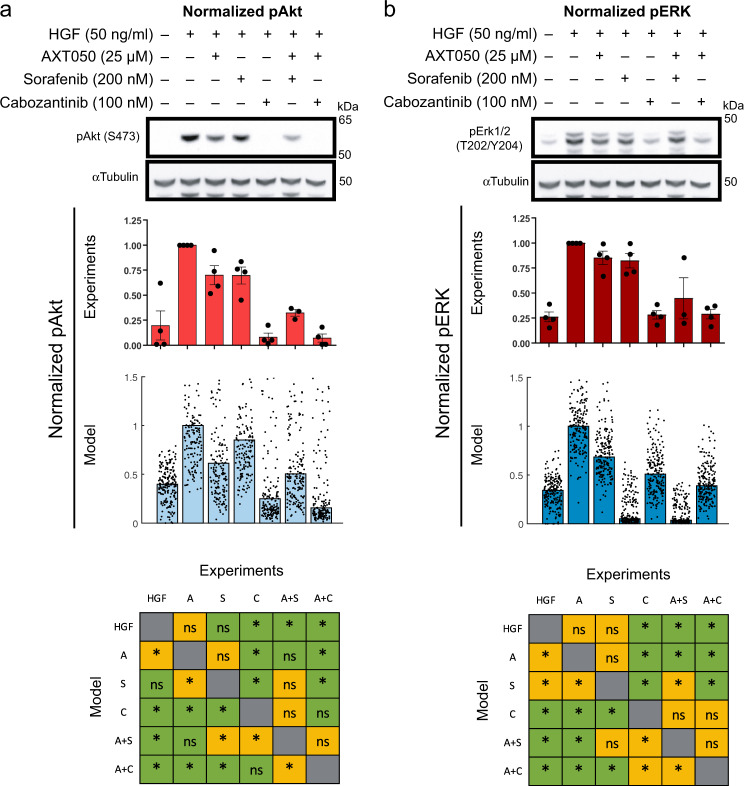
Experimental data and comparison with model. Phosphorylation of Akt (**a**) and ERK (extracellular-regulated kinase) (**b**) and were measured for hepatocyte growth factor (HGF) treatment along with AXT050, sorafenib and cabozantinib or AXT050 + sorafenib and AXT05 + cabozantinib. Cropped images of the western blots and the experimental data (blue) were normalized to HGF control for each gel and is shown as mean ± SEM (*n* = 4, except for AXT050 + sorafenib with *n* = 3). Uncropped images of the sample blots are available in the supplements (Supplementary Fig. 6). The modeling results are shown as median (bar graph) and the data points for all the Monte Carlo resampled cases. Experimental data were compared using analysis of variance with Tukey’s multiple comparisons correction, while non-parametric Kruskal–Wallis test with Bonferroni correction was used to compare modeled groups. Significance was assumed at corrected *p* < 0.05. Bottom row compares the significant changes between model and experiments

Our model was validated by comparing the simulation results to experimental data obtained by this work (Fig. 3) and independent experimental data from the literature (Supplementary Fig. 2). As a first step, we performed experiments on HepG2 cells, in which pAkt and pERK were measured after 120 min under mono-treatment with AXT050, sorafenib, and cabozantinib, as well as combination treatment using AXT050 with either sorafenib or cabozantinib (Fig. 3). The model predictions captured the trend of pAkt inhibition by both monotherapies and combination treatments (Fig. 3a). In contrast, the model predicted stronger inhibition of pERK by sorafenib compared to cabozantinib (Fig. 3b). This is most likely caused by the lack of dose–response data; this would allow us to more accurately fit the effect of the drug-induced inhibition of pERK. The model was calibrated with only one dose of sorafenib (1000 nM) versus no treatment (Supplementary Fig. 2a) and for cabozantinib we used the reported binding dynamics without having access to the dose–response data. Additionally, the effects of combination of inhibitors were used to test the model validation. The effects of treatment with individual inhibitors of Met, PDK1, MEK, and PI3K on pAKt and pERK and with combinations of Met + PDK1 and MEK + PI3K inhibitors were measured in a previous study.^33^ All the calibrated model parameters were fixed and then only the strength of the inhibitors was fitted to the pAKt and pERK data from individual treatments with inhibitors of Met, PDK1, MEK, and PI3K,^33^ and using these inhibition strength values and the fitted model we predicted the results of combination therapy (Supplementary Fig. 2a). The model captured the effect of treating cells with a combination of inhibitors of Met and PDK1 showing reduced pAkt in early and late timepoints. The effect of this combination on pERK levels was more limited, in agreement with the experimental data. For the combination of inhibitors of MEK and PI3K, the model predicted lower pERK consistent with experiments, but the lowered pAkt predicted by the model was lower than the data from experiments. A second set of data was used to compare the effect of ERK inhibition on pMEK and pERK (Fig. 3b). We simulated different levels of ERK output blockade, and the model could predict an increase in pMEK and reduction in pERK. Overall, these comparisons were used to validate the model qualitatively or semi-quantitatively.

### Identification of the important parameters in HGF signaling

**Fig. 4:**
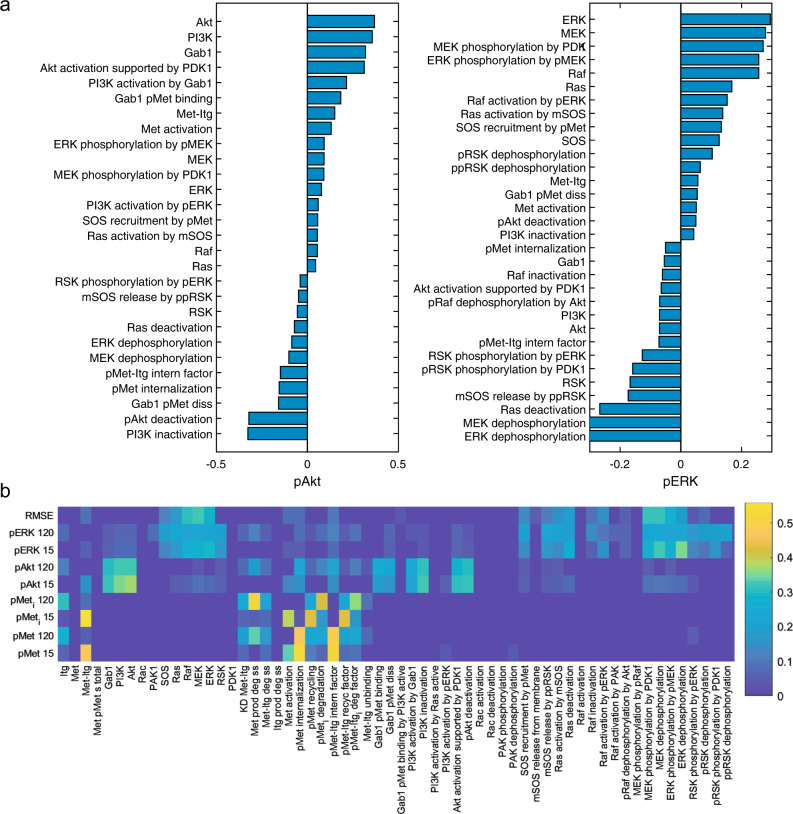
Global parameter sensitivity analysis. Global sensitivity analysis was performed by varying all model parameters simultaneously and performing partial rank correlation analysis to find the effect of inputs (total protein abundance and reactions rates) on the model outputs of interest, primarily phosphorylation of Akt and ERK (extracellular-regulated kinase) at 120 min (**a**). Only the parameters with significant effect (*p* < 0.01) are shown in **a**. Furthermore, the effect of parameters on a larger set of model outputs was explored (**b**) that illustrates localization of parameters that affect the Met receptor dynamics and also crosstalk between the Akt and ERK arms of the pathway. Magnitude of partial rank correlation coefficients (PRCCs) are shown for output parameters and specified at two time points of 15n and 120 min (**b**). All PRCCs are based on the number of molecules per cell as output and only the significant (*p* < 0.01) PRCCs have non-zero values in the heatmap (**b**). Itg in the list of species and parameter names refers to α5β1 integrin

Parameter sensitivity analysis was performed to identify the important parameters affecting the model outputs (Fig. 4). Both reaction rates and protein abundances (inputs) were varied using Latin hypercube sampling (LHS), and partial rank correlation coefficients (PRCCs)^36^ were calculated and significant PRCCs reported for pAkt and pERK (outputs) at 120 min that represents steady state (Fig. 4a). The top candidates positively affecting steady-state levels of pAkt were abundance of Akt, abundance of PI3K, and abundance of GAB1, while PI3K inactivation, pAkt deactivation, and Gab1 pMet dissociation were the top parameters negatively affecting the steady-state levels of pAkt (Fig. 4a). Among notable feedback and crosstalk parameters are PI3K activation by pERK and pERK dephosphorylation that positively and negatively modulate pAkt, respectively (Fig. 4a). For steady-state levels of pERK, the top ranked positive regulators were abundance of ERK, MEK, and the phosphorylation of MEK by PDK1, whereas the most important negative regulators were ERK dephosphorylation, MEK dephosphorylation, and Ras deactivation (Fig. 4a). Based on pERK sensitivity analysis data, activation of Akt pathway strongly and negatively modulates pERK. This analysis can be performed for any other output of interest within the scope of the model. Effects of parameter variation on early (15 min) and late (120 min) pAkt, pERK, internalized pMet (pMet_i_), surface pMet (pMet), and root mean square error of the fit were explored and reported using heatmaps (Fig. 4b). The outputs of pMet and pMet_i_ were only affected by parameters directly modulating Met or α5β1 integrin, evident by the cluster of parameters in the bottom middle section of the heatmap (Fig. 4b). The interrelated top section of the heatmap demonstrates the crosstalk between the Akt and ERK sides of the pathway with the parameters on one side affecting the outputs on the other side and vice versa (Fig. 4b).

### Targeting α5β1 integrin with ECM-derived mimetic peptide AXT050 is a rational strategy

**Fig. 5:**
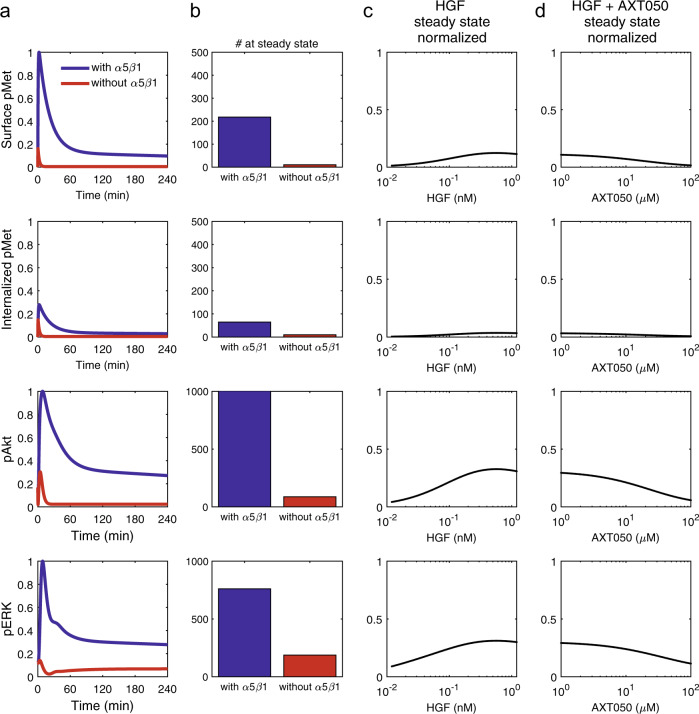
Integrin is important in hepatocyte growth factor (HGF)/Met signaling and AXT050 efficiently blocks the Met/α5β1 interaction (**a**). The model predicts that the response without α5β1 contribution meaning the condition that HGF (40 ng/ml) only signaled through Met and not Met-α5β1 (red) is considerably lower than when Met is allowed to interact with α5β1 (blue). The quantitation of the total number of phosphorylated proteins in a cell showed phosphorylated AKT (pAkt) levels to be more dependent on Met/α5β1 interaction than pERK and pMet levels (**b**). Most of the pMet signal (73%) was from pMet_i_ signaling form endosomes compared to surface pMet (second row compared to first row). Increase in HGF concentration boosted steady-state pAkt and pERK response, but the transient phosphorylation peaks at earlier timepoints were higher in magnitude (**c**). AXT050 treatment efficiently blocked pAkt with pERK being less affected by the treatment (**d**)

The model predicts that HGF stimulation primarily signals through internalized receptors. The contribution of the pMet_i_ was 73% of total pMet in a cell that resulted in about a quarter of the signal through surface pMet (Fig. 5a, first versus second row—blue lines). To investigate the role of α5β1 integrin, in Met signaling, we compared the baseline case that included α5β1 (Fig. 5a—blue lines) with a scenario in which the same number of Met receptors are present without any α5β1 interaction (Fig. 5a—red lines). It is important to note that the activation rate of Met upon binding to HGF is assumed to be independent of the α5β1 binding, but the rates of internalization, degradation, and recycling of the receptor were assumed to be dependent on α5β1 binding. The model predicts that regulation of Met trafficking through α5β1 binding is an important regulator of downstream Akt and ERK signaling (Fig. 5a, b). In particular, Akt phosphorylation, compared to ERK phosphorylation, appeared to be more sensitive to the phosphorylation of Met, which is evident by the dramatic reduction in pAkt in the case without α5β1 (Fig. 5b). As expected, stimulation with higher concentrations of HGF increased both the surface pMet and the internalized pMet, as well as pAkt and pERK (Fig. 5c). Furthermore, the model reproduces the experimental data that peptide binding to α5β1 integrin resulted in reduced phosphorylation of Met, Akt, and ERK (Figs. 2c and 5d). These findings suggest that the ECM-derived mimetic peptide, AXT050, is a logical strategy for targeted therapy.

### The model predicts synergistic efficacy for combination of AXT050 with sorafenib, cabozantinib, and rilotumumab but not synergistic potency

**Fig. 6:**
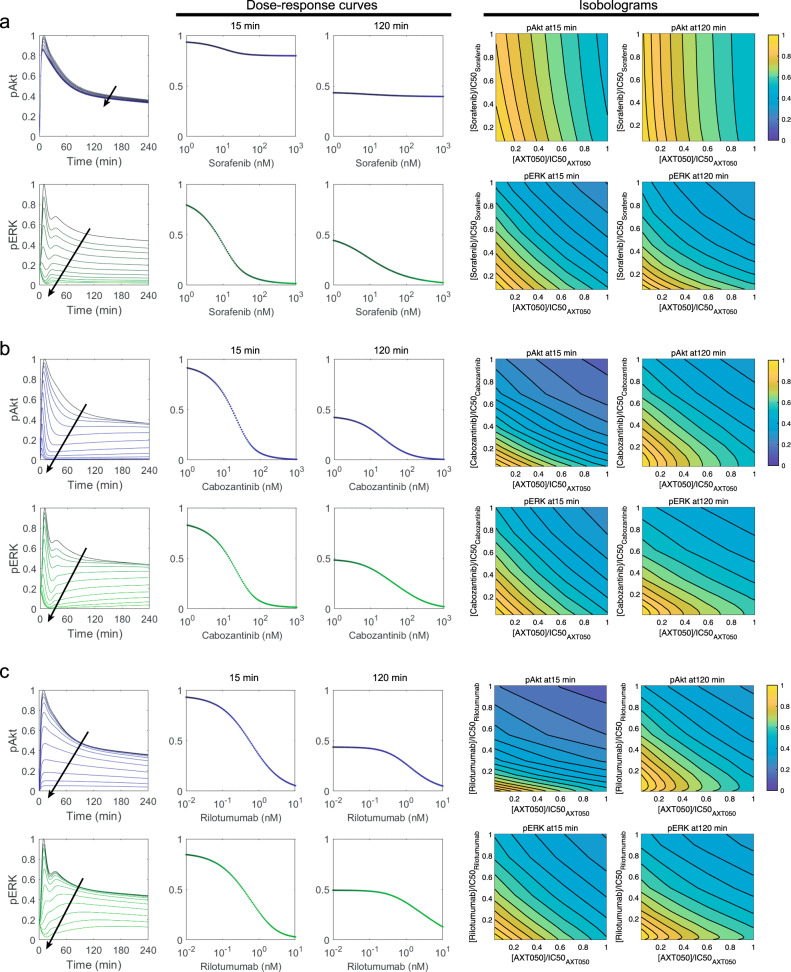
Combination of AXT050 peptide with other drugs targeting the hepatocyte growth factor (HGF) pathway. Combination of AXT050 peptide with sorafenib (**a**), cabozantinib (**b**), and rilotumumab (**c**) along with their respective isobolograms are illustrated here. In the first column, arrows show the direction of increase in the inhibitor concentration, which was varied in the range of 1–1000 nM for sorafenib, 1–1000 nM for cabozantinib, and 0.01–10 nM for rilotumumab. Phosphorylation of Akt (blue) and ERK (extracellular-regulated kinase) (green) are shown for simulations of different levels of each inhibitor (first column). Dose-dependent changes in Akt and ERK phosphorylation at early timepoint of 15 min (second column) and late timepoint of 120 min (third column) illustrates the inhibition potential of each simulation drug as monotherapy. The combination of AXT050 peptide with each of the drugs was simulated over the therapeutic range, half-maximal inhibitory concentrations (IC_50_s) were calculated, and isobolograms were produced for 15 min (fourth column) and 120 min (fifth column) for phosphorylated Akt and ERK, to be able to visually explore synergistic combinations. All simulations are under 40 ng/ml HGF treatment

We next used the calibrated and validated model to investigate the effect of the combination of AXT050 with other therapeutics targeting HGF/Met signaling pathway to predict synergy. The primary outputs of pAkt and pERK were calculated at early (15 min) and late (120 min) timepoints. The later timepoint is a representative measure of the model output at steady state. Combinations of the novel therapeutic peptide, AXT050, with three drugs: sorafenib—a Raf inhibitor (Fig. 6a), cabozantinib—a Met inhibitor (Fig. 6b), and rilotumumab—an HGF inhibitor (Fig. 6c) were considered. Dose–response curves illustrated how sorafenib treatment alone reduced pERK in both early and late timepoints with almost complete blockade of pERK at steady state (Fig. 6a), which follows the trends in the calibration dataset used at 30 min. Sorafenib inhibited pAkt at the early timepoint, the steady-state pAkt only showed very slight decrease. Quantification of the synergy using multidimensional synergy of combinations (MuSyC)^37^ technique for AXT050 and sorafenib at steady state suggest an inconsequential synergistic efficacy (*β*_obs_ = 0.001 and 0.035 for pAkt and pERK, respectively), as well as a negligible antagonistic potency (log(*α*_2_) = −0.02 and −0.05 for pAkt and pERK, respectively). Cabozantinib monotherapy inhibited both pAkt and pERK at the early and late timepoints with a more efficient inhibition of pAkt. MuSyC suggests an unimportant synergistic efficacy (*β*_obs_ = 0.005 and 0.001 for pAkt and pERK, respectively), but antagonistic potency (log(*α*_2_) = −0.68 and −0.35 for pAkt and pERK, respectively) for AXT050 and cabozantinib at steady state. The effect of rilotumumab was implemented by reducing the HGF concentration based on depletion of HGF through binding to rilotumumab based on the binding characteristics of this antibody. Similar to the previous cases, quantification of the synergy by MuSyC for AXT050 and rilotumumab at steady state revealed an inconsequential synergistic efficacy (*β*_obs_ = 0.013 and 0.007 for pAkt and pERK, respectively) and antagonistic potency (log(*α*_2_) = −0.31 and −0.10 for pAkt and pERK, respectively). Monotherapy with rilotumumab had similar effect to that of cabozantinib with a smaller effect on the levels of pERK at the early timepoint. The combination of AXT050 and rilotumumab showed a synergy at the early timepoint for pAkt and displayed additive behavior for the rest of the outputs.

### A patient-specific model identifies patients who could benefit from monotherapy and synergistic drug combinations

**Fig. 7:**
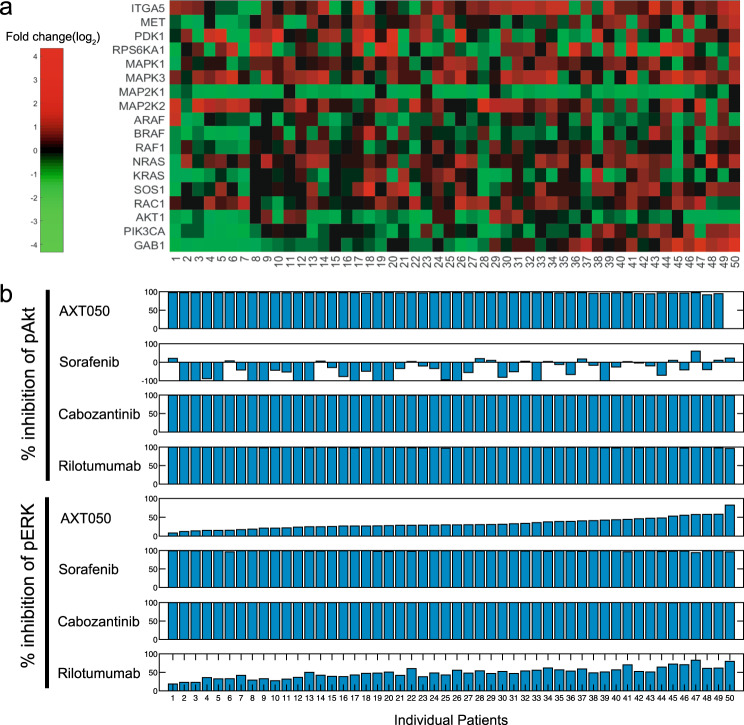
Patient-specific response to monotherapy treatment (**a**). Variations in model proteins were extracted from a cohort of hepatocellular carcinoma (HCC) patients available through The Cancer Genome Atlas (TCGA) (top panel: log_2_ of fold change in RNA level, *n* = 50) (**b**). Percent inhibition of the levels of phosphorylated Akt (pAkt) and pERK was quantified for every patient under each of the four treatments studied here. Each column shows the data for a patient from TCGA, and they are sorted based on the effect of AXT050 on pERK and are aligned for both **a** and **b**

We then employed the model to investigate the utility of monotherapy and combination treatments for individual patients based on the TCGA data. Messenger RNA (mRNA) level data on the proteins in the HGF/Met pathway were extracted from the database and for the tumor and healthy tissue samples for each patient (Fig. 7a). Because the majority of the calibration datasets were from healthy hepatocytes, we assume that the model represents the healthy liver cells of the patient. Hence, individual simulated patients were created by keeping the reaction rates constant while scaling the abundance of proteins based on the fold change in the mRNA of each protein in the tumor compared to the same patient’s healthy tissue. Simulations of monotherapy showed that all the drugs studied here except sorafenib were able to completely reduce the levels of pAkt at steady state (Fig. 7b). In some cases, the model showed that sorafenib increases the pAkt at steady state, a result that is also observed experimentally.^38,39^ Sorafenib and cabozantinib almost completely depleted the pERK signal, while AXT050 and rilotumumab showed inhibition in a range of 8–82% and 18–84%, respectively, across the patients.

**Fig. 8:**
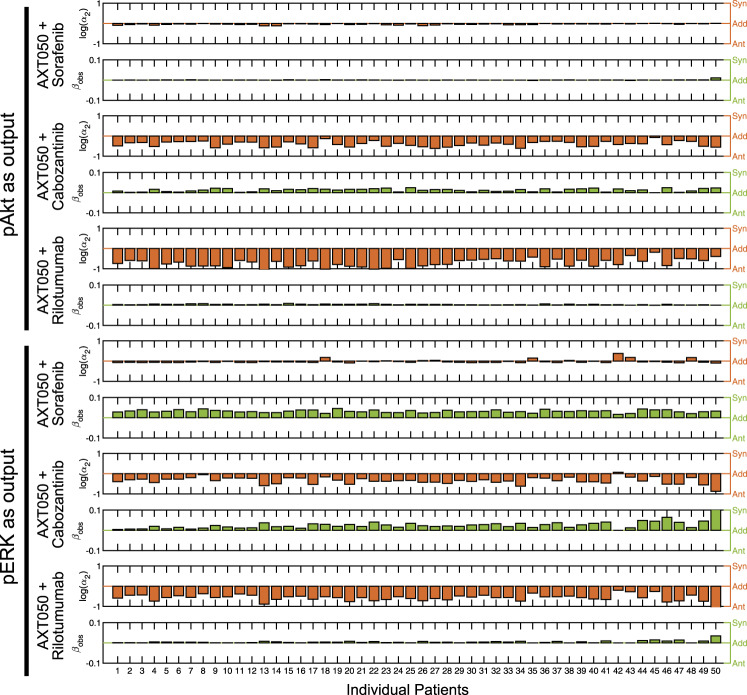
Patient-specific response to combination treatment. Assuming phosphorylation of Akt or ERK (extracellular-regulated kinase) as outputs, the synergistic potency (log(*α*_2_) > 0) and synergistic efficacy (*β*_obs_ > 0) were calculated to measure the synergy between the peptide (AXT050) and three drugs against the HGF pathway (sorafenib, cabozantinib, and rilotumumab). Log(*α*_2_) > 0 shows synergistic potency and *β*_obs_ > 0 shows synergistic efficacy. Each column shows the data for a patient from TCGA ordered the same as patients in Fig. 7

We then investigated the effect of combination therapy on the levels of pAkt and pERK response in individual patients simulated (Fig. 8). Synergy in efficacy (*β*_obs_ > 0) as well as potency (log(*α*_2_) > 0) were quantified using MuSyC technique that was introduced by Meyer et al.^37^ and is described in the Methods. For pAkt as output, combinations of AXT050 with each of the other three drugs (sorafenib, cabozantinib, and rilotumumab) showed negligible synergistic efficacy, but antagonistic potency. In particular, combination of AXT050 with cabozantinib and rilotumumab were highly antagonistic most likely due to the fact that they all target the activity at the receptor level. Similarly, for pERK as the output, all three combinations showed modest synergistic efficacy (<10% additional efficacy). Combination of AXT050 and sorafenib showed a minor synergistic potency in ~20% of the patients, whereas its combination with cabozantionib and rilutumumab was mostly antagonistic for the TCGA patients studied here. To compare the similarity of HCC cell lines to the TCGA patients, we correlated mRNA levels of 373 TCGA HCC samples and three liver cancer cell lines from Broad Institute Cancer Cell Line Encyclopedia (HuH7, Hep3B217, and HepG2) and created a heatmap of Spearman’s correlation coefficients (Supplementary Fig. 5). The cell lines showed a similar expression profile to that of primary tumors, although a comprehensive study is necessary to compare the similarity of these cell lines to HCC patients versus other tumor types.

## Discussion

The HGF/Met pathway is often proposed as a mechanism of resistance to other kinase inhibitors such as inhibitors of VEGF and EGF pathways. We developed a model of HGF/Met signaling pathways connected to important intracellular signals of growth and survival (i.e., Akt and ERK), which included many identified feedback loops and crosstalk between these two critical cellular regulators. The model included a detailed representation of the Met interaction with α5β1 integrin to be able to investigate the effect of the integrin-targeting peptide, AXT050. This model was then calibrated using data from multiple sources previously published in the literature for hepatocytes and HCC cell lines^30,33,34^ and was validated for use in HCC. We identified the important model parameters affecting the phosphorylation of Akt and ERK using parameter sensitivity analysis, and showed the dynamics of AXT050 inhibition of HGF/Met signaling. Furthermore, effect of monotherapy as well as combination therapy was investigated using the calibrated model. The model was then extended to simulate individual patients based on the data gathered from TCGA to investigate the efficacy of monotherapy and combination therapy of a number of drugs explored in this study.

Association of Met and α5β1 integrin has been shown to be important in regulation of growth and survival pathways.^21–26,30^ This model of the HGF/Met signaling pathway was able to capture the contribution of the Met-α5β1 association, and allowed us to investigate the effect of the α5β1-binding peptide, AXT050, on pAkt and pERK. Our previous work has shown that in vitro treatment of HepG2 cells with AXT050 resulted in inhibition of pMet, pAkt, and pERK,^30^ as well as other RTKs such as VEGR2, IGFR, and PDGFR.^30^ The simulations in this study confirmed the experimental results and showed that efficient inhibition of the pAkt an pERK at steady state was primarily due to dissociation of Met from α5β1, which in turn resulted in a shift in response to inefficient phosphorylation of Met alone without integrin association. The model predicted that regulation of receptor trafficking (internalization, degradation, and recycling) rates through α5β1 association is sufficient to explain the experimentally observed data. Furthermore, the prediction of the model on dominant HGF/Met signaling through internalized receptors is similar to the experimental data acquired for other receptors such as VEGFR2.^40^ AXT050 has also been shown to inhibit tumor growth in vivo in a Myc-induced syngeneic mouse model of HCC and also in a mouse HepG2 tumor xenograft model.^30^ Although AXT050 could reduce the growth of the HCC cells in the tumor, the primary effect of this drug is thought to be its antiangiogenic capabilities that mitigate tumor growth.^30^ In the future studies, recalibration of this model for endothelial cells and integration with previously developed models of the VEGF pathway^31,41–43^ would allow us to better dissect the contribution of the antiangiogenic effect versus the direct effect of AXT050 on HCC cells in HCC tumors.

Sorafenib has been the standard of care for unresectable HCC since its approval in 2007.^10^ In recent years, approvals of the new TKIs (lenvatinib as the first line and regorafenib and cabozantinib as the second line) and immune checkpoint blocking antibodies (nivolumab and pembrolizumab, both blocking PD-1) have energized the field to seek novel targets and drug combinations for HCC treatment. Our model showed that AXT050 was synergistic in combination with cabozantinib and rilotumumab, both of which target interaction of HGF and Met. However, we should also note a possible limitation of the model in that the sole representation of HGF/Met signaling pathway in the HCC cells may not be sufficient to capture the multimodal effect of these drugs *in vivo*. For example, both AXT050 and cabozantinib exhibit an antiangiogenic effects in addition to Met inhibition, which is not considered in this version of the model. One of the limitations of this study was the lack of dose–response data on pMet for calibration of the effect of rilotumumab. The binding affinity of the antibody was used to represent this anti-HGF antibody. Rilotumumab antibody studied here was a sample antibody and is currently less significant clinically, as further development of this antibody was halted due to increased mortality.^15^ However, other antibodies targeting HGF are in development, such as YYB101.^44^ In addition to antibodies directly targeting HGF, there are multiple antibodies targeting Met activation, notably MM-131 from Merrimack, which is a bispecific anti-Met/EpCAM that inhibits Met activation through HGF-dependent and -independent paths.^45^

The HGF/Met signaling pathway is often thought of as one of the primary mechanisms of resistance to EGFR inhibitors and antiangiogenic therapies.^46^ EGFR is one of the most active growth factors that maintains tumor growth and survival in carcinomas. Blockade of EGFR often leads to secondary activating mutations in EGF-family receptors or overexpression of Met.^47^ High levels of HGF are correlated with poor response to EGFR inhibitors in colon cancer as well as in non-small-cell lung cancer.^48^ This effect is more pronounced in HCC, as the healthy liver utilizes HGF/Met signaling for regeneration, thus having a higher basal concentration of HGF in the liver and the tumor microenvironment in HCC.^6^ Inhibition of Met in combination with cetuximab (monoclonal antibody that inhibits EGFR) has been shown to overcome the EGFR resistance, although many of these patients exhibit resistance from other sources.^49^ Expansion of this model to include other RTK signaling pathways such as EGFR, VEGFR, and fibroblast growth factor receptor would allow us to investigate mechanisms of resistance to monotherapy as well as assist us in identifying efficacious combinations based on individual HCC patient data.^50^ A model of EGFR-Met crosstalk was implemented by Shin et al.^50^ to investigate the synergy in anti-EGFR and anti-Met therapies. Furthermore, Hass et al.^51^ developed a multi-pathway model of tyrosine kinase signaling of EGFR, HER2, ErbB3, Met, and IGF1R down to ERK and Akt and characterized the variability of ligand-induced response based on the RNA expression levels of the ligands across TCGA patients. Several models of HGF/Met pathway have been published before.^33,52–54^ Meyer et al.^52^ developed a model of HGF-mediated Akt activation and demonstrated that heterogeneity of the response between cells could only be explained by variability in concentration of various proteins. From another direction, Boolean models of HGF pathway along other tyrosine kinase pathways and tumor growth factor-β (TGFβ) have been used to investigate the EMT in HCC.^53^ Other continuum models of HGF and TGFβ have been developed to study the crosstalk between cancer cells and cancer-associated fibroblasts in tumor microenvironment.^54^ This study builds upon the previous models by developing a dynamic model of HGF/Met signaling to Akt and ERK in HCC cells and patient-specific tumors and explores the synergy of a novel α5β1 integrin-binding therapeutic (AXT050) with other drugs targeting this important pathway.

To implement the effect of particular drugs on the outputs of the pathway, we used the reported binding rates for interaction of the drug with its target. Our model predicted half-maximal inhibitory concentration (IC_50_) of the drugs with respect to pAkt and pERK. The model predicted sorafenib IC_50_ to be ~200 nM for pERK, which was lower than the previously published in vitro data showing sorafenib IC_50_ to be 3.2–10 μM for pERK in a variety of cancer cell lines.^55^ The higher in vitro IC_50_s are associated with the serum content in the experimental protocols.^55^ The increase in steady-state pAkt with sorafenib treatment was unexpected at first, but a complete literature search revealed that similar responses had been measured experimentally before.^39^ In the clinic, HCC patients receive a 400 mg dose of sorafenib every 12 h, leading to an average plasma concentration of 6.2 μg/ml,^56^ which is well above the concentration of 40 ng/ml often used in experiments and in this study.

To build confidence in our model, we followed the standard practices of system pharmacology modeling^57,58^ and the model credibility framework suggested by the Committee on Credible Practice of Modeling and Simulation in Healthcare^59^ in Interagency Modeling and Analysis Groupestablished by National Institutes of Health (NIH) and other federal agencies. The context of the model was defined as HGF/Met signaling in advanced HCC, and we elected to only use the relevant data from hepatocytes or HCC cell lines. Although the goal was to represent human HCC, we had no choice but to use the time-course immunoblots^33^ and proteomic data^35^ from mouse primary hepatocytes. Indeed, a recent proteomic study showed that 92% of proteins in adipose-derived mesenchymal stem cells of mouse and human are consistent.^60^ We also added minimal details to the interactions of Met and α5β1 to be able to study the effect of AXT050 that targets and breaks the interaction of α5β1 with Met. HGF-independent activation of Met from binding of α5β1 to fibronectin^22^ was neglected in this model due to lack of sufficient experimental data, but could be implemented in the future. Another limitation of the model was the use of mRNA to scale the calibrated model for individual patients, which was due to lack of data on the protein levels for the proteins of interest in TCGA. A similar assumption was made by Shin et al.^50^ to model RTK signaling in breast cancer patients. Acceptable and statistically significant correlations have been shown between mRNA and protein levels for some of the proteins of the pathway that had both measurements available on TCGA by this study (Supplementary Fig. 4) and previous works.^50^ A simplifying assumption made was that the baseline model represents healthy tissue (due to the fact that most of the calibration data came from healthy hepatocytes) for all the healthy patients, with the understanding that there are interindividual variations that are not captured by the model. In the future, addition of dose–response curves for the effect of drugs on the outputs of interest could further improve prediction capabilities of the model. Implementation of the likelihood methods could be beneficial in the future iterations of the model to improve efficiency of the confidence interval calculations.^61,62^ Additionally, we assumed that the tumor is homogenous, which is often not the case for tumors, especially for HCC tumors in advanced stages.^63^ Agent-based models could be combined with this model to investigate the effect of spatial distribution of cells and growth factors on the tumor response.^64–66^ We used a Git server on the local network for version control and local sharing. The final working model in Systems Biology Markup Language (SBML) format is provided in the supplemental materials to facilitate reusability of the model.

In summary, we developed a model of HGF/Met signaling pathway to Akt and ERK with details of interactions with α5β1 integrin, which was calibrated and validated with a consistent set of experimental data from hepatocytes and human HCC cell lines that allowed us to better understand the mechanism of action of AXT050 and other HGF pathway-targeting therapeutics. The simulations provided us with insight on the mechanism of action of AXT050 and allowed us to study the differences in efficacy of monotherapy and combination therapy for individual patients based on TCGA data. With the expected increase in the availability of patient-specific genomic data in the future, an expanded version of this model that includes multiple parallel pathways in cancer cells and tumor stromal cells could benefit individual patients through identification of combination therapies that have higher probability of exhibiting efficacy for the individual patient.

## Methods

### Computational model structure

Our model included interactions of HGF with surface receptor Met and α5β1 integrin, as well as the downstream intracellular signaling pathways that lead to phosphorylation of Akt and ERK, the two major signals crucial for survival and proliferation (Fig. 1). Synthesis, internalization, and degradation were incorporated for the surface molecules (Met and α5β1 integrin). The integrin-binding therapeutic peptide (AXT050) was assumed to bind α5β1 integrin and dissociate it from Met, thereby depleting α5β1 integrin heterodimer. The intracellular molecules are assumed to have constant total protein numbers and to switch between the active form (often phosphorylation modification) and the inactive form. The details of the intracellular pathway are based on a previous model of HGF signaling in hepatocytes by D’Alessandro et al.,^33^ which utilized logic approaches to ensure optimal inclusion of feedback loops among all identified biological mechanisms. In contrast to the previous model that treated all the species concentrations or copy numbers normalized between 0 and 1, we completely re-parameterized the model to include the total copy number of each protein measured through an extensive proteomic study in mouse primary hepatocytes.^35^ Sorafenib inhibition strength was fitted directly to experimental data. Cabozantinib was assumed to bind Met based on its relevant binding affinity and using first-order reaction kinetics. Rilotumumab was modeled by reducing the extracellular HGF concentration based on the assumption of having the drug in excess. The rest of the inhibitors used in the model calibration were modeled based on previous work by reducing the signal going out of the node based on an inhibition parameter.^33^ The model was developed using the SimBiology platform in MATLAB R2018b (MathWorks) and all the simulations and sensitivity analyses were done in MATLAB. This model included 52 species and 69 parameters including all the inhibitors modeled here (Supplementary Tables 1–4). The model used for this study is provided in the Supplementary material using SBML format (Supplementary File 1).

### Model calibration and validation

The pattern search algorithm in MATLAB’s global optimization toolbox was used to simultaneously fit all the model parameters.^31,41–43^ An extensive time-course immunoblot data provided in ref. ^33^ were used as the primary data for calibration (Fig. 2a). Phosphorylation of Met, Akt, MEK, ERK, and RSK were measured in primary mouse hepatocytes treated with HGF alone or in combination with MEK inhibitor, PDK1 inhibitor, or both. PDK1 is a master kinase that primarily regulates Akt activation^67^ and contributes to MEK activation.^68^ These two inhibitors were chosen in the original experiments to allow dissection of the relative importance of different feedback loops and crosstalk in the HGF/Met pathway. Furthermore, we used a second set of previously published data from our laboratory that measured the effect of treatment with various concentrations of AXT050 on phosphorylation of Met, Akt, and ERK in HepG2 (human HCC cell line) (Fig. 2b).^30^ Additionally, we utilized the data from Melas et al.,^34^ who reported the level of phosphorylation of Akt, MEK, and ERK as average response from three HCC cell lines (Huh7, Hep3b, and HepG2) at 30 min after treatment compared to pre-treatment, to fit the strength of inhibition of Raf by sorafenib (Supplementary Fig. 2a). The internalized proportion of Met was also fitted to another set of data that measured 50% internalization at 15 min^69^ (Supplementary Fig. 2b). A qualitative validation was performed based on the additional data from this study and also combination treatments reported in ref. ^33^ Phosphorylation of Akt and ERK after mono-treatment with inhibitors of Met, PDK1, MEK, and PI3K as well as the combination of Met + PDK1 and MEK + PI3K inhibitors have been reported. While fixing the model parameters that were calibrated with the time-course data (Fig. 2), pAkt and pERK data from mono-treatment with individual inhibitors were used to fit the strength of these inhibitors, and the combination simulations were compared to the combination data (Supplementary Fig. 3a). Moreover, phosphorylation of MEK and ERK was compared to the data on ERK inhibition from the same study (Supplementary Fig. 3b).

### Uncertainty quantification and identifiability

To quantify the uncertainty in our parameter estimation, we performed a Monte Carlo resampling from a log-normal distribution similar to the methods used by others.^70,71^ Briefly, we used parametric resampling assuming a log-normal distribution about the mean of each time point to obtain 201 resampled datasets; we assumed a 10% measurement error to obtain an estimate of the population standard deviation as in previous studies.^61^ Each dataset was used to re-estimate the model parameters to determine the distribution of the parameter estimates. Model fitting was done using the pattern search from global optimization toolbox in MATLAB with the parameters estimated from the original dataset as the initial fit; the original fit plus-or-minus one order of magnitude was used as bounds for the optimization. This procedure was also used to determine the effect of the parameter uncertainty on the model results by obtaining a distribution of simulations. The model contains 56 free parameters that were globally fitted. For visualization, the 95% confidence interval of the distribution of solutions obtained by Monte Carlo resampling was calculated and shown in the figures as a shaded region surrounding the solution obtained with the original parameterization. Finally, local parameter sensitivities to important model outputs (pMet, pAkt, pMEK, pERK, pRSK, and ppRSK) were calculated for each of the new fits to obtain a distribution of sensitivities. In this study, we defined a parameter to be practically identifiable if 95% of its distribution of sensitivities to at least one of the outputs maintains a consistent sign as done in previous studies.^70–73^

### Parameter sensitivity analysis

To better understand the effect of model parameters on the outputs of the model and identification of the impactful parameters, a standard parameter sensitivity analysis was performed. LHS was used to simultaneously vary all model parameters uniformly within ±50% range to investigate the effect of model inputs on phosphorylation of Akt and ERK. Five thousand cases were simulated and PRCCs as well as the *p* values for each correlation were calculated based on the previously published method.^36^ In the bar graphs, only parameters with significant (*p* < 0.01) PRCCs are shown (Fig. 4a), and in the heatmaps the insignificant (*p* ≥ 0.01) PRCCs are replaced with zero (Fig. 4b).

### Data extraction from TCGA database

RNA sequencing level 3 RSEM normalized data for HCC^74^ from TCGA were accessed from the Broad Institute TCGA GDAC Firehose (https://ezid.cdlib.org/id/doi:10.7908/C11G0KM9) and log 2 transformed. We used 50 match tumor-normal pair to calculate pairwise fold change in expression.

### Quantification of synergy

Synergy of combination therapy was assessed using MuSyC technique for the baseline case and individual patients.^37^ Briefly, a surface was fitted to the two-dimensional dose space of the two drugs of interest and two parameters representing synergistic potency (log(*α*_2_) > 0) and synergistic efficacy (*β*_obs_ > 0) were quantified for each case (Fig. 8). *β*_obs_ works similar to *E*_max_ and *α*_2_ is analogous to half-maximal effective concentration (EC50) in traditional single drug pharmacology. Values of *β*_obs_ shows fold change in efficacy due to the added combination, while log(*α*_2_) that is on base 10 shows order-of-magnitude change in potency. In this study, we have presented the data as combination of other three drugs with AXT050.

### Cell culture and peptide handling

The HCC line HepG2 (American Type Culture Collection (ATCC) Manassas, VA, USA) was maintained in Dulbecco’s modified Eagle’s media (DMEM) with 4.5 g/l glucose (Corning, Corning, NY), 10% fetal bovine serum (Corning), and 100 U/ml penicillin and streptomycin (Gibco). These cells were authenticated using the GenePrint 10 kit (Promega) to obtain a short tandem repeat (STR) profile, which was then compared to the ATCC STR database. Mycoplasma contamination was not tested. AXT050 was produced by solid-phase synthesis and purchased from New England Peptide. High-performance liquid chromatography and mass spectrometry analysis indicated a purity >90%. For working solutions, the lyophilized peptide was dissolved in 100% DMSO to a concentration of 40 mM and stored at −20 °C until used. For cell-based experiments, aliquots were diluted to 2 mM working stocks in water. Excess dimethyl sulfoxide (DMSO) was added to each culture to normalize the final DMSO concentration to 0.06% in all samples.

### Western blots

HepG2 cells were seeded into 6-well plates coated with 10 μg/ml fibronectin (Sigma) and cultured for 48 h in full serum DMEM media at 37 °C and 5% CO_2_. The cells were then serum starved overnight in serum-free DMEM media. The next day, cultures were treated with 25 μM AXT050 or DMSO vehicle for 90 min, followed by treatment with either 200 nM sorafenib tosylate (ChemScene, Monmouth Junction, NJ) or 100 nM cabozantinib malate (APExBIO, Houston, TX) or DMSO vehicle. Cells were then immediately stimulated with 50 ng/ml HGF for 120 min. Cells were then transferred to ice, washed twice with cold Dulbecco’s phosphate-buffered saline, and lysed in 180 μl SDS Loading Dye (Cell Signaling Technologies, Danvers, MA). Lysates were then sonicated, boiled, and stored at −20 °C until needed. Lysates were resolved by sodium dodecyl sulfate-polyacrylamide gel electrophoresis using 4–12% gradient NuPAGE gel in MOPS buffer (Life Technologies) and transferred to nitrocellulose membranes for Western blotting. Membranes were blocked in 5% bovine serum albumin (BSA) (Sigma-Aldrich, St. Louis, MO) and 5% milk (LabScientific Inc., Highlands, NJ) and incubated overnight with the following primary antibodies in TBST (Tris-buffered saline, 0.1% Tween-20) containing 5% BSA and 0.03% sodium azide: Cell Signaling—pAkt (S473) (Cat#: 4058), pERK1/2 (T202/Y204)) (Cat#: 4370), glyceraldehyde 3-phosphate dehydrogenase (Cat#: 91766); Abcam—α-tubulin (Cat#: ab4074). Bands were detected by chemiluminescence using horse radish peroxidase-conjugated secondary goat anti-rabbit and sheep anti-mouse antibodies (Cell Signaling) diluted in 5% milk in TBST with either the ChemiDoc (Bio-Rad) and the associated Image Lab software or KwikQuant (Kindle Biosciences) imaging systems. Densitometry analysis was performed using ImageJ software (NIH). Western blot experiments were repeated four times using different cell passages or stocks for each experiment. One sample in the AXT050 and sorafenib co-treatment group was excluded owing to an experimental artifact that prevented accurate quantification. For analysis, replicate blots were each derived from the same experiment and processed in parallel.

### Statistical analysis

All western blot experiments were completed at least four separate times. The means for the normalized, relative phosphorylation from each treatment group were compared to each other by analysis of variance followed by Tukey’s multiple comparisons test using GraphPad Prism® software v.5.0. Non-parametric Kruskal–Wallis test with Bonferroni multi-comparison correction was used to compare the groups in modeling results and the analysis was performed in MATLAB R2018b (MathWorks). A *p* value <0.05 was considered significant.

### Reporting summary

Further information on research design is available in the Nature Research Reporting Summary linked to this article.

## Supplementary information

Supplementary material

Reporting Summary

## Data Availability

All data generated or analyzed during this study as well as the model in SBML format are included in this published article and its supplementary materials.

## Supplementary information

**Supplementary Information** accompanies the paper on the *npj Systems Biology and Applications* website (10.1038/s41540-019-0107-2).

## Code availability

The model is provided in the standard SBML format as Supplementary File 1.
