# Supplementary material for: Mechanistically detailed systems biology modeling of the HGF/Met pathway in hepatocellular carcinoma
Source: NPJ Syst Biol Appl. 2019 Aug 16;5:29. doi: 10.1038/s41540-019-0107-2 (PMC6697704; doi:10.1038/s41540-019-0107-2)
Supplement: Supplementary file 1 — Supplementary material [file 41540_2019_107_MOESM1_ESM.docx]

**Mechanistically-detailed Systems Biology Modeling of the HGF/Met Pathway in Hepatocellular Carcinoma**

# Mohammad Jafarnejad, Richard J. Sove, Ludmila Danilova, Adam C. Mirando, Yu Zhang, Mark Yarchoan, Phuoc T. Tran, Niranjan B. Pandey, Elana J. Fertig, Aleksander S. Popel

**Supplementary Figures**


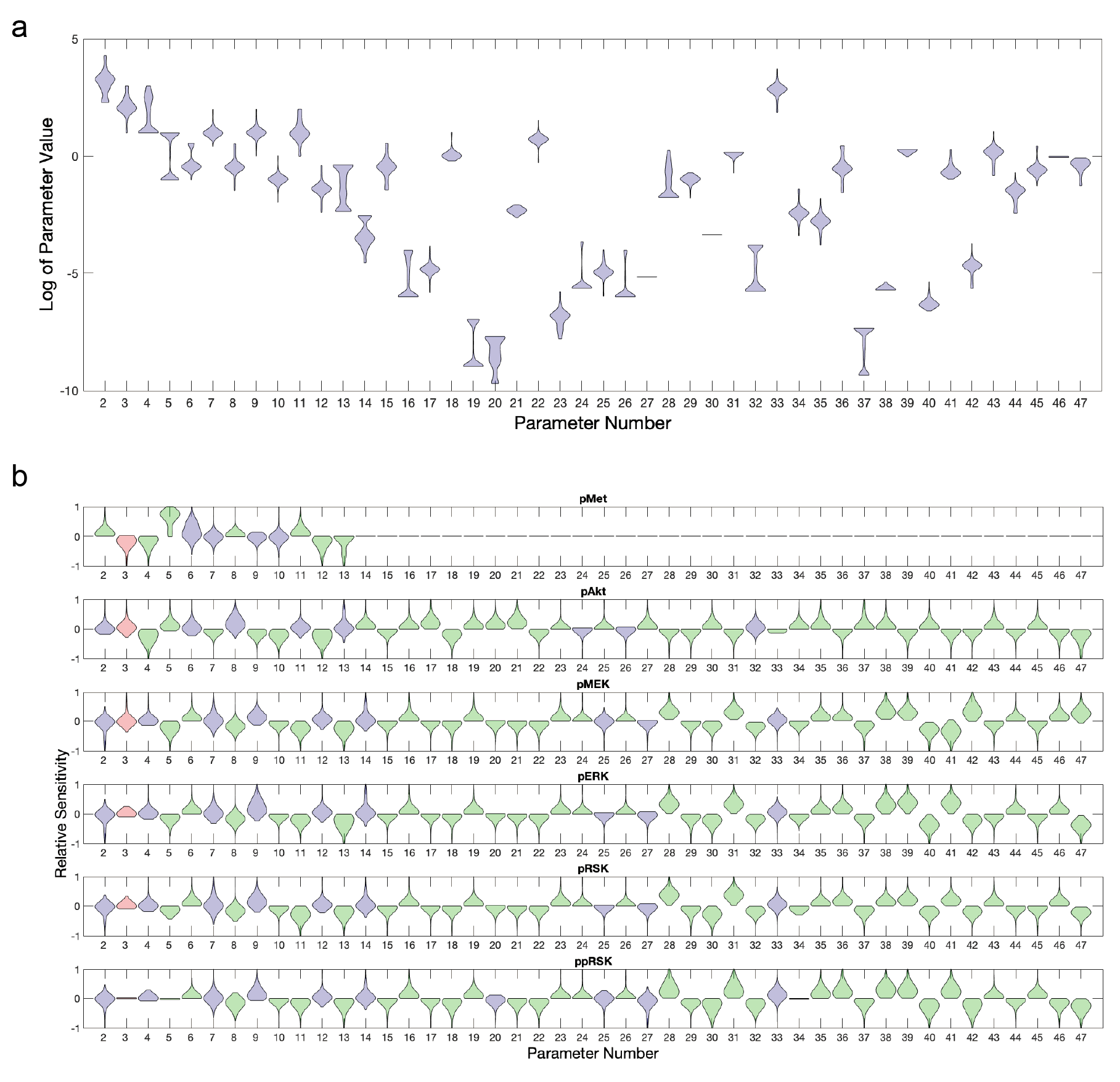


Supplementary Figure 1. Model identifiability.

a. Probability density function of the model parameters from the bootstrap simulations showing the distribution of parameters. b. Sensitivity of the outputs (top to bottom: pMet, pAkt, pMEK, pERK, pRSK, and ppRSK) shows the parameters that are identifiable across all outputs (green), the parameters that are identifiable for at least one of the outputs (blue), and the one that is unidentifiable (red). Only parameter 3 (basal production and degradation of Met) was unidentifiable. The parameter numbers shown here correlate with parameter information presented in Supplementary Table 2.


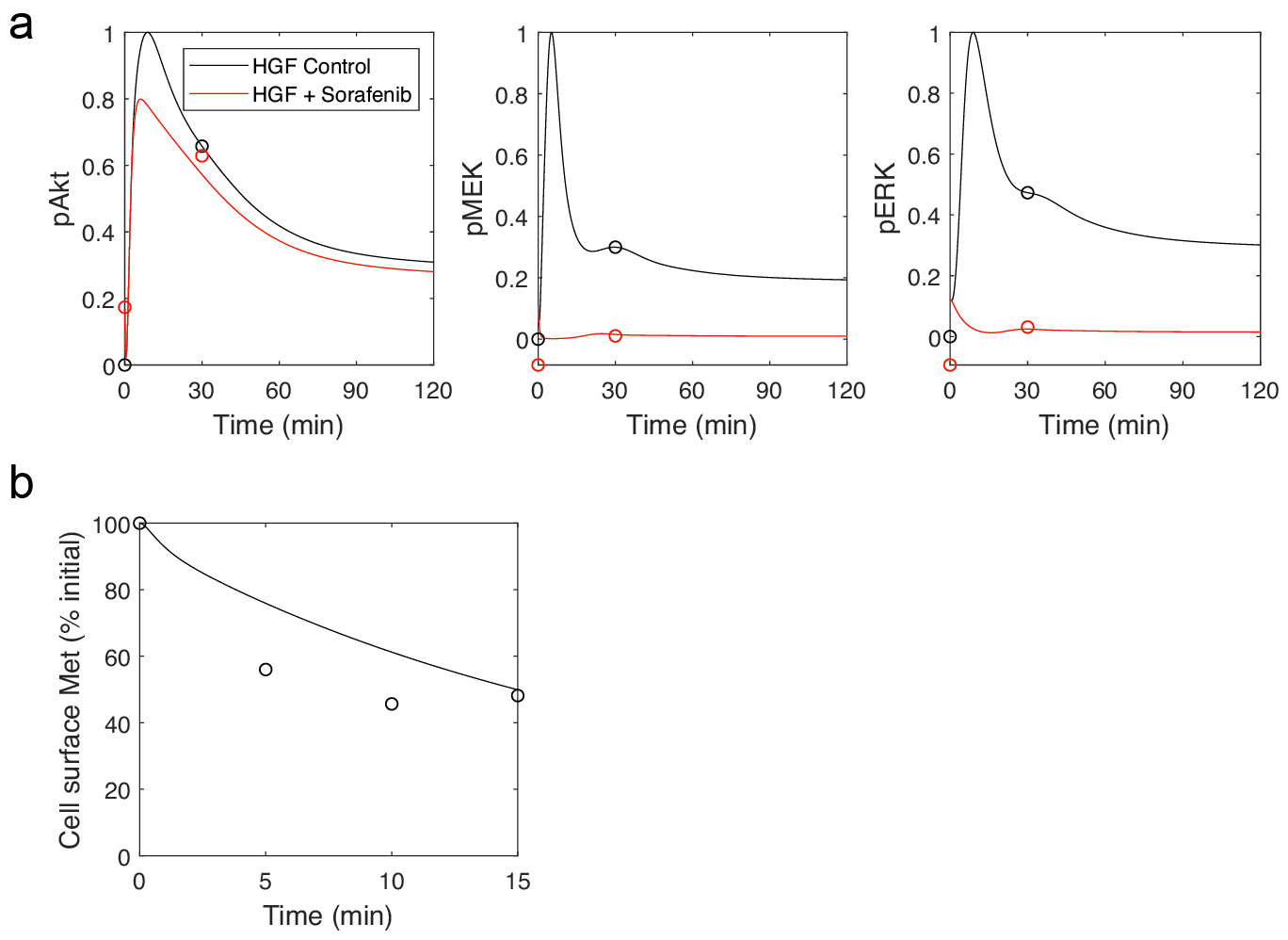


Supplementary Figure 2. Additional Model calibration data.

a. The magnitude of sorafenib inhibition was fitted to a dataset available from Melas et al. (PMID: 25729777) reported as average response from three HCC cell lines (Huh7, Hep3b, and HepG2) at the 30 min timepoint (n = 1 for each cell line, but averaged over three HCC cell lines). b. Internalized pMet was also fitted to the experimental data from Li et al. (n = 1 for the available data, PMID: 19066037).


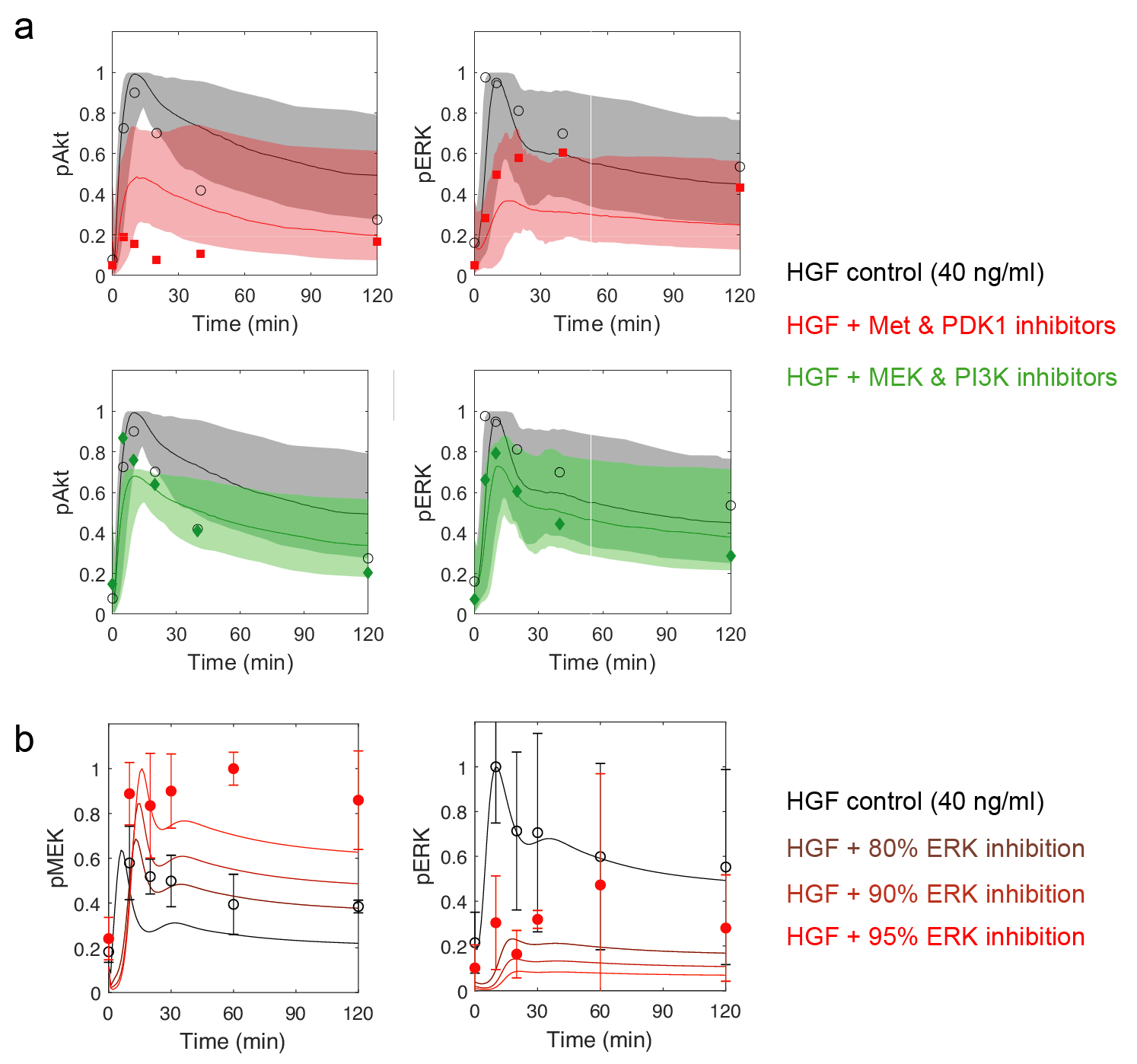


Supplementary Figure 3. Additional model validation.

The model was validated using additional data reported in the literature [^46^](#_ENREF_46). a. The magnitude of inhibition by individual inhibitors of Met, PDK1, MEK and PI3K was fitted to the data from individual inhibition experiments (not shown here), and then the phosphorylation of Akt and ERK from combination therapies was compared to the model outputs (n = 1). b. Additionally, phosphorylation of MEK and ERK was compared for different levels of ERK inhibition from the same study (mean ± SD ,n = 3). ERK affects MEK through a positive feedback loop to Raf and an indirect negative feedback loop from ppRSK to SOS (Figure 1). Model correctly predicts that inhibition of ERK leads to overall increase in pMEK signal.


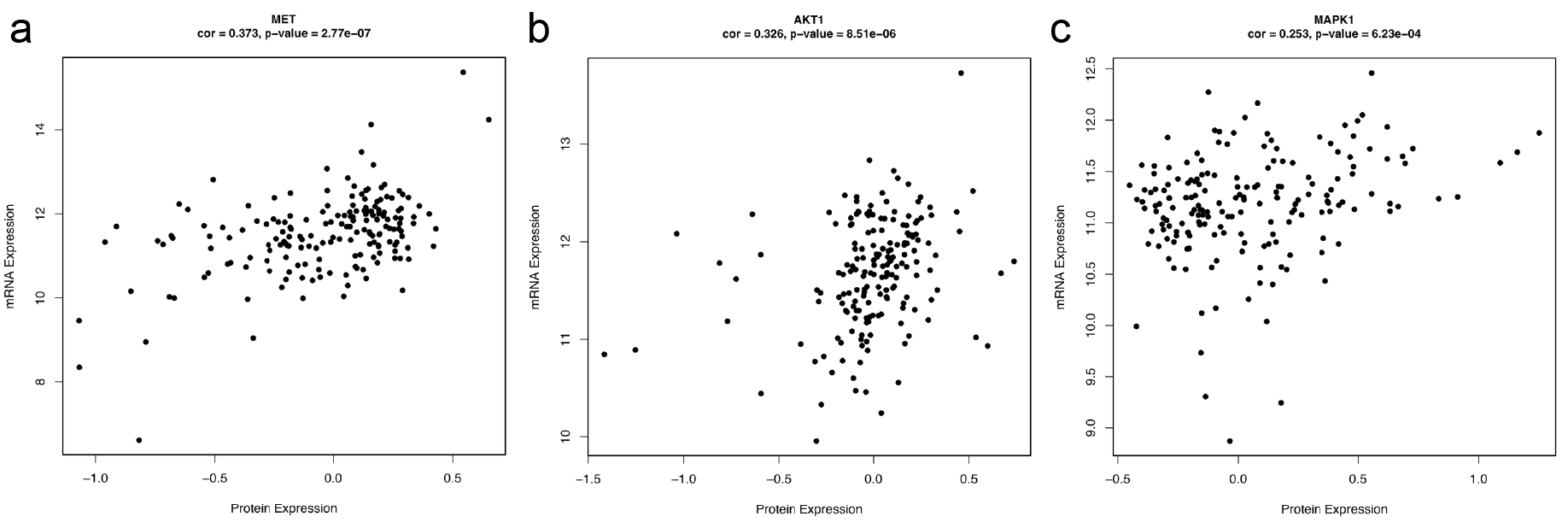


Supplementary Figure 4. Correlation of RNA and protein based on TCGA data.

mRNA and protein levels of MET, AKT, and ERK were correlated in 181 TCGA hepatocellular carcinoma cancer samples that had both RNAseq and RPPA data available. Protein levels of MET (a), AKT (b), and ERK (c) are very correlated with their mRNA levels (Spearman’s correlation coefficients are 0.37, 0.33, and 0.25, respectively).


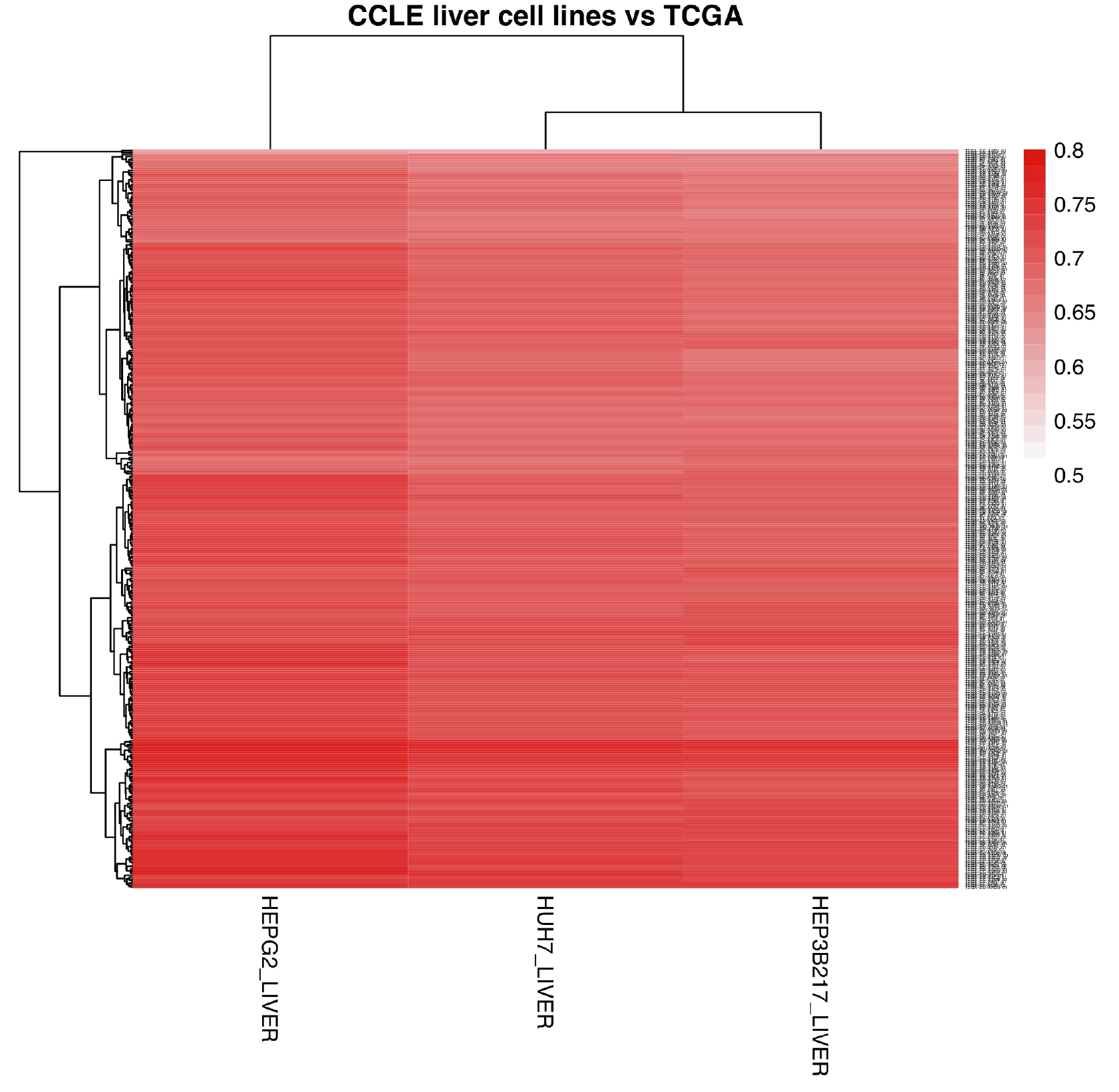


Supplementary Figure 5. Additional model validation.

Spearman’s correlation coefficients for 373 TCGA hepatocellular carcinoma samples and three liver cancer cell lines from Broad Institute Cancer Cell Line Encyclopedia (HuH7, Hep3B217, and HepG2) showing that the cell lines have similar expression profile to primary tumors.


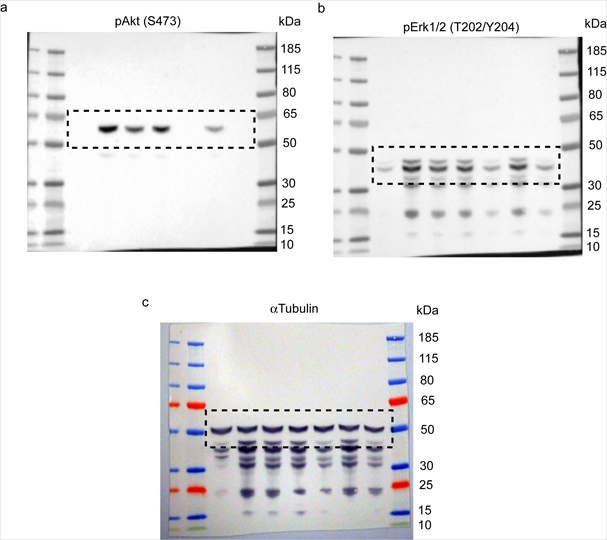


Supplementary Figure 6. Uncropped Western blot images with molecular weight markers.

Uncropped versions of the Western blot images included in Figure 3 with a white light overlay to show molecular weight markers. Western blots were stained serially in the following order: pAkt (S473) (a), pErk1/2 (T202/Y204) (b), and αTubulin (c). Dotted lines indicate the estimated boundaries of the cropped components used in Figure 3.

**Supplementary Tables**

Supplementary Table 1. Species used in the model.

| Species name | Initial amount | Compartment |
| --- | --- | --- |
| Itg | 15527 | Hepatocyte |
| Met | 9 | Hepatocyte |
| Met_Itg | 6590 | Hepatocyte |
| pMet | 0 | Hepatocyte |
| pMet_i | 0 | Hepatocyte |
| pMet_Itg | 0 | Hepatocyte |
| pMet_Itg_i | 0 | Hepatocyte |
| pMet_total | 0 | Hepatocyte |
| pMet_i_total | 0 | Hepatocyte |
| Met_pMet_s_total | 6600 | Hepatocyte |
| pMet_s_total | 0 | Hepatocyte |
| Gab1 | 5914 | Hepatocyte |
| Gab1_pMet | 0 | Hepatocyte |
| PI3K | 1976 | Hepatocyte |
| PI3K_active | 0 | Hepatocyte |
| Akt | 32983 | Hepatocyte |
| pAkt | 0 | Hepatocyte |
| Rac | 731654 | Hepatocyte |
| Rac_active | 0 | Hepatocyte |
| PAK1 | 2000 | Hepatocyte |
| pPAK1 | 0 | Hepatocyte |
| SOS | 6948 | Hepatocyte |
| mSOS_pMet | 0 | Hepatocyte |
| Ras | 30826 | Hepatocyte |
| Ras_active | 0 | Hepatocyte |
| Raf | 89608 | Hepatocyte |
| pRaf | 0 | Hepatocyte |
| MEK | 259694 | Hepatocyte |
| pMEK | 0 | Hepatocyte |
| ERK | 232907 | Hepatocyte |
| pERK | 0 | Hepatocyte |
| RSK | 70636 | Hepatocyte |
| pRSK | 0 | Hepatocyte |
| ppRSK | 0 | Hepatocyte |
| PDK1 | 56563 | Hepatocyte |
| Peptide_Itg | 0 | Hepatocyte |
| Sorafenib_Raf | 0 | Hepatocyte |
| Cabozantinib_Met | 0 | Hepatocyte |
| Cabozantinib_Met_Itg | 0 | Hepatocyte |
| Met_max | 9454 | Hepatocyte |
| Itg_max | 51270 | Hepatocyte |
| Akt_max | 32983 | Hepatocyte |
| Raf_max | 89608 | Hepatocyte |
| MEK_max | 259694 | Hepatocyte |
| ERK_max | 232907 | Hepatocyte |
| RSK_max | 70636 | Hepatocyte |
| HGF | 0 | Tumor |
| Peptide | 0 | Tumor |
| Sorafenib | 0 | Tumor |
| Cabozantinib | 0 | Tumor |

Supplementary Table 2. Model parameters.

| ID | Parameter name | Value | Unit |
| --- | --- | --- | --- |
| 1 | KD_HGF_Met | 0.025 | nM |
| 2 | KD_Met_Itg | 1975.376 | molecule |
| 3 | Met_prod_deg_ss | 100 | molecule s^-1 |
| 4 | Met_Itg_deg_ss | 100 | molecule s^-1 |
| 5 | Itg_prod_deg_ss | 1 | molecule s^-1 |
| 6 | Met_activation | 0.364046 | nM^-1 s^-1 |
| 7 | pMet_internalization | 9.79571 | s^-1 |
| 8 | pMet_recycling | 0.342383 | s^-1 |
| 9 | pMet_i_degradation | 10 | s^-1 |
| 10 | pMet_Itg_intern_factor | 0.10753 | dimensionless |
| 11 | pMet_Itg_recyc_factor | 9.999978 | dimensionless |
| 12 | pMet_Itg_i_deg_factor | 0.040611 | dimensionless |
| 13 | Met_Itg_unbinding | 0.044066 | s^-1 |
| 14 | Gab1_pMet_binding | 0.000297 | molecule^-1 s^-1 |
| 15 | Gab1_pMet_diss | 0.364726 | s^-1 |
| 16 | Gab1_pMet_binding_by_PI3K_active | 0.00001 | molecule^-1 s^-1 |
| 17 | PI3K_activation_by_Gab1 | 1.49E-05 | molecule^-1 s^-1 |
| 18 | PI3K_inactivation | 1.047623 | s^-1 |
| 19 | PI3K_activation_by_Ras_active | 1.1E-08 | molecule^-1 s^-1 |
| 20 | PI3K_activation_by_pERK | 2.02E-09 | molecule^-1 s^-1 |
| 21 | Akt_activation_supported_by_PDK1 | 0.004949 | molecule^-1 s^-1 |
| 22 | pAkt_deactivation | 5.326702 | s^-1 |
| 23 | Rac_activation | 1.58E-07 | molecule^-1 s^-1 |
| 24 | Rac_deactivation | 2.32E-05 | s^-1 |
| 25 | PAK_phosphorylation | 1.05E-05 | molecule^-1 s^-1 |
| 26 | PAK_dephosphorylation | 0.00001 | s^-1 |
| 27 | SOS_recruitment_by_pMet | 6.88E-06 | molecule^-1 s^-1 |
| 28 | mSOS_release_from_membrane | 0.178008 | s^-1 |
| 29 | mSOS_release_by_ppRSK | 0.109027 | molecule^-1 s^-1 |
| 30 | Ras_activation_by_mSOS | 0.000459 | molecule^-1 s^-1 |
| 31 | Ras_deactivation | 1.506371 | s^-1 |
| 32 | Raf_activation | 1.68E-05 | molecule^-1 s^-1 |
| 33 | Raf_inactivation | 725.4092 | s^-1 |
| 34 | Raf_activation_by_pERK | 0.004144 | molecule^-1 s^-1 |
| 35 | Raf_activation_by_PAK | 0.001646 | molecule^-1 s^-1 |
| 36 | pRaf_dephosphorylation_by_Akt | 0.284073 | s^-1 |
| 37 | MEK_phosphorylation_by_pRaf | 4.6E-09 | molecule^-1 s^-1 |
| 38 | MEK_phosphorylation_by_PDK1 | 1.92E-06 | molecule^-1 s^-1 |
| 39 | MEK_dephosphorylation | 1.975952 | s^-1 |
| 40 | ERK_phosphorylation_by_pMEK | 4.36E-07 | molecule^-1 s^-1 |
| 41 | ERK_dephosphorylation | 0.196965 | s^-1 |
| 42 | RSK_phosphorylation_by_pERK | 2.24E-05 | molecule^-1 s^-1 |
| 43 | pRSK_dephosphorylation | 1.535599 | s^-1 |
| 44 | pRSK_phosphorylation_by_PDK1 | 0.037926 | molecule^-1 s^-1 |
| 45 | ppRSK_dephosphorylation | 0.277526 | s^-1 |
| 46 | MEK_inhib | 0.927106 | dimensionless |
| 47 | PDK_inhib | 0.570295 | dimensionless |
| 48 | ERK_inhib | 1 | dimensionless |
| 49 | Akt_inhib | 1 | dimensionless |
| 50 | PI3K_inhib | 1 | dimensionless |
| 51 | Met_inhib | 1 | dimensionless |
| 52 | KD_Peptide_Itg | 99.27186 | nM |
| 53 | Peptide_Itg_unbinding | 0.000107 | s^-1 |
| 54 | Met_Itg_diss_peptide | 0.008937 | s^-1 |
| 55 | KD_Sorafenib | 5.632727 | nM |
| 56 | Sorafenib_unbinding | 0.073581 | s^-1 |
| 57 | KD_Cabozantinib | 1.3 | nM |
| 58 | Cabozantinib_unbinding | 0.01 | s^-1 |
| 59 | KD_Rilotumumab | 0.22 | nM |
| 60 | Met_scale | 1 | molecule |
| 61 | Itg_scale | 1 | molecule |
| 62 | Akt_scale | 0.089066 | molecule |
| 63 | Raf_scale | 0.492365 | molecule |
| 64 | MEK_scale | 0.021995 | molecule |
| 65 | ERK_scale | 0.006316 | molecule |
| 66 | pRSK_scale | 0.025376 | molecule |
| 67 | ppRSK_scale | 0.00229 | molecule |

Supplementary Table 3. Model reactions.

| Reaction | Reaction rate |
| --- | --- |
| null -> Hepatocyte.Met | Met_prod_deg_ss |
| Hepatocyte.Met -> null | Met*Met_prod_deg_ss/Met_max |
| Hepatocyte.Met_Itg -> null | Met_Itg*Met_Itg_deg_ss/Met_max |
| Hepatocyte.Itg + Hepatocyte.Met -> Hepatocyte.Met_Itg | Itg*Met*Met_Itg_unbinding/KD_Met_Itg |
| Hepatocyte.Met_Itg -> Hepatocyte.Itg + Hepatocyte.Met | Met_Itg*Met_Itg_unbinding |
| Tumor.HGF + Hepatocyte.Met_Itg -> Hepatocyte.pMet_Itg | HGF*Met_Itg*Met_activation |
| Hepatocyte.pMet_Itg -> Hepatocyte.Met_Itg | pMet_Itg*KD_HGF_Met*Met_activation |
| Tumor.HGF + Hepatocyte.Met -> Hepatocyte.pMet | HGF*Met*Met_activation |
| Hepatocyte.pMet -> Hepatocyte.Met | pMet*KD_HGF_Met*Met_activation |
| Hepatocyte.pMet -> Hepatocyte.pMet_i | pMet*pMet_internalization |
| Hepatocyte.pMet_Itg -> Hepatocyte.pMet_Itg_i | pMet_Itg*pMet_internalization*pMet_Itg_intern_factor |
| Hepatocyte.pMet_i -> Hepatocyte.Met | pMet_i*pMet_recycling |
| Hepatocyte.pMet_Itg_i -> Hepatocyte.Met_Itg | pMet_Itg_i*pMet_recycling*pMet_Itg_recyc_factor |
| Hepatocyte.pMet_i -> null | pMet_i*pMet_i_degradation |
| Hepatocyte.pMet_Itg_i -> null | pMet_Itg_i*pMet_i_degradation*pMet_Itg_i_deg_factor |
| null -> Hepatocyte.Itg | Itg_prod_deg_ss |
| Hepatocyte.Itg -> null | Itg*Itg_prod_deg_ss/Itg_max |
| Hepatocyte.Itg + Tumor.Peptide -> Hepatocyte.Peptide_Itg | Itg*Peptide* Peptide_Itg_unbinding/KD_Peptide_Itg |
| Hepatocyte.Peptide_Itg -> Hepatocyte.Itg + Tumor.Peptide | Peptide_Itg*Peptide_Itg_unbinding |
| Hepatocyte.Met_Itg + Tumor.Peptide -> Hepatocyte.Itg + Hepatocyte.Met + Tumor.Peptide | Met_Itg *Peptide * Met_Itg_diss_peptide |
| Hepatocyte.pMet_Itg + Tumor.Peptide -> Hepatocyte.Itg + Hepatocyte.Met + Tumor.Peptide | pMet_Itg *Peptide * Met_Itg_diss_peptide |
| Hepatocyte.Gab1 -> Hepatocyte.Gab1_pMet | Gab1*Gab1_pMet_binding*(pMet + pMet_i + pMet_Itg + pMet_Itg_i)*(1-Met_inhib) |
| Hepatocyte.Gab1_pMet -> Hepatocyte.Gab1 | Gab1_pMet*Gab1_pMet_diss |
| Hepatocyte.Gab1 -> Hepatocyte.Gab1_pMet | Gab1*Gab1_pMet_binding_by_PI3K_active*PI3K_active*pMet |
| Hepatocyte.PI3K -> Hepatocyte.PI3K_active | Gab1_pMet*PI3K*PI3K_activation_by_Gab1 |
| Hepatocyte.PI3K_active -> Hepatocyte.PI3K | PI3K_active*PI3K_inactivation |
| Hepatocyte.PI3K -> Hepatocyte.PI3K_active | PI3K*PI3K_activation_by_Ras_active*Ras_active |
| Hepatocyte.PI3K -> Hepatocyte.PI3K_active | Gab1_pMet*PI3K*PI3K_activation_by_pERK*pERK |
| Hepatocyte.Akt -> Hepatocyte.pAkt | Akt*Akt_activation_supported_by_PDK1*PI3K_active*(1-PI3K_inhib) |
| Hepatocyte.pAkt -> Hepatocyte.Akt | pAkt*pAkt_deactivation |
| Hepatocyte.Rac -> Hepatocyte.Rac_active | Gab1_pMet*Rac*Rac_activation |
| Hepatocyte.Rac_active -> Hepatocyte.Rac | Rac_active*Rac_deactivation |
| Hepatocyte.PAK1 -> Hepatocyte.pPAK1 | PAK1*PAK_phosphorylation*Rac_active |
| Hepatocyte.pPAK1 -> Hepatocyte.PAK1 | PAK_dephosphorylation*pPAK1 |
| Hepatocyte.SOS -> Hepatocyte.mSOS_pMet | SOS*SOS_recruitment_by_pMet*(pMet + pMet_i + pMet_Itg + pMet_Itg_i)*(1-Met_inhib) |
| Hepatocyte.mSOS_pMet -> Hepatocyte.SOS | mSOS_pMet*mSOS_release_from_membrane |
| Hepatocyte.mSOS_pMet -> Hepatocyte.SOS | ppRSK*mSOS_pMet*mSOS_release_by_ppRSK |
| Hepatocyte.Ras -> Hepatocyte.Ras_active | Ras*Ras_activation_by_mSOS*mSOS_pMet |
| Hepatocyte.Ras_active -> Hepatocyte.Ras | Ras_active*Ras_deactivation |
| Hepatocyte.Raf -> Hepatocyte.pRaf | Raf*Raf_activation*Ras_active |
| Hepatocyte.pRaf -> Hepatocyte.Raf | Raf_inactivation*pRaf |
| Hepatocyte.Raf -> Hepatocyte.pRaf | Raf*Raf_activation_by_pERK*Ras_active*pERK*(1-ERK_inhib) |
| Hepatocyte.Raf -> Hepatocyte.pRaf | Raf*Raf_activation_by_PAK*Ras_active*pPAK1 |
| Hepatocyte.pRaf -> Hepatocyte.Raf | pAkt*pRaf*pRaf_dephosphorylation_by_Akt*(1-Akt_inhib) |
| Hepatocyte.MEK -> Hepatocyte.pMEK | MEK*MEK_phosphorylation_by_pRaf*pRaf |
| Hepatocyte.pMEK -> Hepatocyte.MEK | MEK_dephosphorylation*pMEK |
| Hepatocyte.MEK -> Hepatocyte.pMEK | MEK*MEK_phosphorylation_by_PDK1*(1-PDK_inhib)*pRaf |
| Hepatocyte.ERK -> Hepatocyte.pERK | ERK*ERK_phosphorylation_by_pMEK*(1-MEK_inhib)*pMEK |
| Hepatocyte.pERK -> Hepatocyte.ERK | ERK_dephosphorylation*pERK |
| Hepatocyte.RSK -> Hepatocyte.pRSK | RSK*RSK_phosphorylation_by_pERK*pERK*(1-ERK_inhib) |
| Hepatocyte.pRSK -> Hepatocyte.RSK | pRSK_dephosphorylation*pRSK |
| Hepatocyte.pRSK -> Hepatocyte.ppRSK | pRSK_phosphorylation_by_PDK1*(1-PDK_inhib)*pRSK |
| Hepatocyte.ppRSK -> Hepatocyte.RSK | ppRSK_dephosphorylation*ppRSK |
| Hepatocyte.Raf + Tumor.Sorafenib -> Hepatocyte.Sorafenib_Raf | Raf * Sorafenib * Sorafenib_unbinding/KD_Sorafenib |
| Hepatocyte.Sorafenib_Raf -> Hepatocyte.Raf | Sorafenib_unbinding * Sorafenib_Raf |
| Hepatocyte.Met + Tumor.Cabozantinib -> Hepatocyte.Cabozantinib_Met | Met * Cabozantinib * Cabozantinib_unbinding/KD_Cabozantinib |
| Hepatocyte.Cabozantinib_Met -> Hepatocyte.Met | Cabozantinib_unbinding * Cabozantinib_Met |
| Hepatocyte.Met_Itg + Tumor.Cabozantinib -> Hepatocyte.Cabozantinib_Met_Itg | Met_Itg * Cabozantinib * Cabozantinib_unbinding/KD_Cabozantinib |
| Hepatocyte.Cabozantinib_Met_Itg -> Hepatocyte.Met_Itg | Cabozantinib_unbinding * Cabozantinib_Met_Itg |

Supplementary Table 4. Model rules.

| Algebraic rule | Rule Type |
| --- | --- |
| pMet_total = pMet + pMet_i + pMet_Itg + pMet_Itg_i | Repeated Assignment |
| pMet_i_total = pMet_i + pMet_Itg_i | Repeated Assignment |
| Met_pMet_s_total = Met + Met_Itg + pMet + pMet_Itg | Repeated Assignment |
| pMet_s_total = pMet + pMet_Itg | Repeated Assignment |
